# Psoriasis and Schizophrenia: An immunological link

**DOI:** 10.1192/j.eurpsy.2024.1422

**Published:** 2024-08-27

**Authors:** B. Fonseca Silva, P. Felgueiras, Â. Pinto, A. Oliveira

**Affiliations:** Centro Hospitalar Vila Nova de Gaia Espinho, Vila Nova de Gaia, Portugal

## Abstract

**Introduction:**

Schizophrenia has progressively been seen as a multifactorial disease, with its pathogenesis including immune dysfunction. Studies have leaned into the activation of brain inflammation, influencing the development of schizophrenia in certain subgroups of patients. Additionally, the role of the T helper (Th17) cells and neuromediators associated are implicated in the pathophysiology of psoriasis, a chronic immune-mediated dermatological condition. A significantly elevated risk was found with 41% increased odds of schizophrenia compared with subjects without psoriasis. The concomitant diagnosis of both illnesses has motivated further investigation into their shared pathways.

**Objectives:**

Characterize the prevalence of psoriasis in patients with schizophrenia and mutual involved mechanisms.

**Methods:**

Retrospective analysis of inpatients of a Portuguese Psychiatry department with the established diagnosis of Schizophrenia, between 2018 and 2022. Additionally a literature review on the topic was conducted.

**Results:**

A sample of 94 patients admitted was obtained. The majority of patients were male (80,1%). The prevalence of the diagnosis of Psoriasis was 6,4% (n=6). A previous epidemiological study conducted in the Portuguese general population concluded that the prevalence of psoriasis is on average 4,4%, which is inferior to the value obtained in our sample. Other studies that measured the relationship between both diagnoses corroborated our results, documenting higher prevalences of psoriasis in patients with schizophrenia than the general population.

**Conclusions:**

The relationship between psoriasis and schizophrenia seems to be bidirectional, with schizophrenia patients having higher risk of psoriasis and psoriasis patients having higher risk of schizophrenia. This could be explained by multiple mechanisms, mainly the activation of Th17 cells but also the fact that there may be a genetic susceptibility due to proximal chromosome loci associated with both diseases (chromosome 6p21.3). This information is essential in providing care to patients because treatment must be carefully adapted. It has been demonstrated that atypical antipsychotics might worsen psoriatic manifestations and immunosuppressive agents are linked to psychotic episodes and worse mental health. Thus, there should be increased alertness for the detection of these conditions in patients with either one of them.

**Disclosure of Interest:**

None Declared

